# 574 The Impact of Age on Wound Healing Progression in Burns

**DOI:** 10.1093/jbcr/irad045.169

**Published:** 2023-05-15

**Authors:** Anna-Lisa Pignet, David Hahn, Anna Schwarz, Manuel Prevedel, Julia Fink, Elisabeth Hofmann, Lars-Peter Kamolz, Petra Kotzbeck, Andrzej Hecker

**Affiliations:** JOANNEUM RESEARCH Forschungsgesellschaft mbH & Medical University of Graz, Graz, Steiermark; Joanneum Research COREMED, Graz, Steiermark; Joanneum Research, Graz, Steiermark; COREMED – Kooperatives Zentrum für Regenerative Medizin, Graz, Steiermark; Joanneum Research, Graz, Steiermark

## Abstract

**Introduction:**

Increasing incidences of wound healing disorders are a growing problem in our aging society. Moreover, the survival rate of the elderly is diminished when it comes to extensive high-grade burn injuries. Here, we characterized the systemic and local reactions towards burn injuries in young, middle aged and aged rats. Additionally, we provide a comparison in wound healing progression between burn wounds and full-thickness wounds of the same sizes.

**Methods:**

For this study, 54 male Wistar rats from three different age groups (11; 27; and 56 weeks) either received high-grade contact burns, full-thickness skin wounds (2x2 wounds, Ø 10 mm each) or served as unwounded controls. Throughout the study regular wound documentation and non-invasive imaging methods were performed. Blood was sampled from the tail vein to detect differences in plasma cytokine concentrations. Moreover, body weight and food intake were measured daily. On the final study day (day 7), tissue biopsies were collected from the wound and control areas and were analyzed on a histological and molecular level. Mainly inflammatory markers (interleukins, TGFb, TNFa etc.) and markers for tissue perfusion (VEGFa, HIF1a) were quantified by qPCR. Repeated measures analysis of variance was performed to compare the means among groups.

**Results:**

We found that epidermal thickness of unwounded skin declined with age and was lowest in 56-weeks-old rats. On day 4 after wounding, tissue perfusion was significantly impaired in 27- and 56-weeks-old rats compared to 11-weeks-old rats (p < 0.001). Histological scores for the immune response showed an age-dependent effect in both burns and excisional wounds. Neutrophil and lymphocyte counts were highest in the 27-weeks-old rats (11 weeks vs. 27 weeks: p < 0.001). Eosinophil counts, however, were significantly increased in burn wounds compared to excisional wounds (p = 0.013), regardless of age. We did not detect any age-dependent effects in wound sizes on the final day, nor in the animals’ weight loss after wounding.

**Conclusions:**

Although there are significant age-related differences in the thickness of the epidermis, the angiogenic potential of the skin and the local immune response, these differences do not seem to affect healing. It is reasonable to assume that decreased wound healing progression occurs only when additional factors, such as impaired perfusion, diabetes etc are involved.

**Applicability of Research to Practice:**

We believe the precise knowledge of processes involved in wound healing is crucial for the development of new therapeutic strategies and to support healing after burns. Our results might challenge the paradigm of impaired healing in aged individuals and draw attention to the necessity to dig for other influential factors. However, as skin morphology and healing dynamics differ between rodents and humans, we plan to implement a translational approach using human abdominal skin flaps of donors from different age groups.

